# Weathering of a Nonwoven Polypropylene Geotextile: Field vs. Laboratory Exposure

**DOI:** 10.3390/ma15228216

**Published:** 2022-11-18

**Authors:** José Ricardo Carneiro, Maria de Lurdes Lopes

**Affiliations:** Construct-Geo, Department of Civil Engineering, Faculty of Engineering, University of Porto, Rua Dr. Roberto Frias, 4200-465 Porto, Portugal

**Keywords:** geosynthetics, geotextiles, durability, weathering, UV-ageing, polypropylene, Chimassorb 944, carbon black

## Abstract

Like other plastic materials, geosynthetics can undergo changes in their properties due to weathering. These changes must be known and, if necessary, duly accounted for in the design phase. This work evaluates the resistance of a nonwoven polypropylene geotextile to weathering, both in the field (under natural degradation conditions) and in the laboratory (under accelerated degradation conditions). The damage experienced by the geotextile in the field weathering tests was evaluated by monitoring changes in its physical (mass per unit area and thickness), mechanical (tensile, tearing and puncture behaviour) and hydraulic (water permeability normal to the plane) properties. Microscopic damage was assessed by scanning electron microscopy. In the laboratory weathering tests, only the tensile behaviour of the geotextile was monitored. The results showed that all geotextile properties were affected by weathering. The mechanical strength of the geotextile decreased in the field weathering tests. Microscopic transverse cracks were found in the weathered polypropylene fibres, which may explain the reduction in mechanical strength. The accumulation of dirt on the nonwoven structure altered the physical and hydraulic properties of the geotextile. Comparing the field and laboratory weathering tests, the reduction in tensile strength found after 24 months outdoors (roughly 30%) was very similar to that observed after 4000 h in the laboratory. This relationship may not be valid for other geotextiles or other exposure locations.

## 1. Introduction

Geosynthetics are construction materials mostly manufactured from thermoplastics, such as polyolefins (e.g., polypropylene (PP)) or polyesters. These materials are not only easy to use but also cost-effective, which, combined with their versatility and good performance over time, makes them suitable materials for many engineering applications. According to their structure, geosynthetics can be divided into different categories, the most common being geotextiles, geomembranes, geogrids and geocomposites. Geotextiles are the most used geosynthetics, being able to perform several functions, including filtration, drainage, separation, protection and reinforcement. The applications of these materials are numerous, having been successfully used, for example, in road infrastructure, erosion control, drainage systems, waste containment and coastal protection structures.

Like other plastic materials, geotextiles are prone to degradation at all stages of their life cycle, including manufacture, storage, installation phase and in-service. These materials typically have high resistance to chemical and biological attack [1], but are susceptible to solar radiation, mainly ultraviolet (UV) radiation [2,3]. The UV zone (≈295 to 400 nm) corresponds to only a small part of the solar radiation reaching the Earth’s surface (≈295 to 3000 nm), but it is the one with the highest energy. Other outdoor degradation agents include heat, oxygen and moisture [4].

Photo-oxidation is the degradation of polymers induced by the combined action of light and oxygen [5,6]. In the case of PP, the oxidation process occurs via an autocatalytic mechanism with initiation, propagation, branching and termination reactions [3,7]. The initiation reaction requires an energy source for the formation of free radicals, which start the mechanism. Energy can be provided by heat (thermo-oxidation) and by UV radiation (photo-oxidation). More information on the oxidation process of PP can be found, for example, in Greenwood et al. [3].

Chemical additives, which are added to polymers during processing, can be used to control the photo-oxidation of plastic materials [5,8]. These compounds can interrupt or slow down the degradation process, being essential to improve the weathering resistance of polymeric products. Examples of effective additives include light stabilisers and antioxidants. As shown in previous works [9,10,11], the weathering resistance of PP geotextiles can be highly enhanced by the presence, in relatively small amounts, of a hindered amine light stabiliser or carbon black.

Predicting the behaviour of geotextiles over time is a complicated process, as it usually requires many aspects to be considered. The predictions are often based on material testing, where they are exposed to degrading conditions. Over the years, standards organisations, such as the European Committee for Standardization or the American Society for Testing and Materials, have developed methods to help evaluate the resistance of geotextiles to degradation. In the case of weathering, the methods developed for geotextiles include EN 12224 [12], ASTM D4355 [13] and ASTM D5970 [14].

Weathering tests can be conducted in the field or in the laboratory. In the field, materials degrade under natural conditions, providing accurate information about their behaviour. The downside is that the tests are usually time-consuming—they can take years [10,11,15]. In the laboratory, artificial weathering tests under accelerated degradation conditions can be carried out in weatherometers. This equipment tries to simulate the degradation suffered by materials exposed outdoors, usually allowing control of the light intensity, temperature and humidity.

Over the years, researchers have evaluated the resistance of geotextiles to weathering by performing field [10,11,15,16,17,18] or laboratory [9,19,20,21] tests. Some works [22,23,24,25] carried out both tests, comparing the results obtained and trying to find relationships between them. The effect of weathering tests on geotextiles is usually evaluated by detecting and quantifying changes in their properties. Tensile properties have been monitored in many works [9,10,11,15,16,17,21,24], being the most used for this purpose. Technics such as microscopy [9,10,15,20,21,24], spectrophotometry [25], infrared spectroscopy [22], thermal analysis [15,20,21] and chromatography [22,23] have also been used to determine the effect of weathering on geotextiles.

The results of previous works have shown that it is practically unavoidable for geotextiles to degrade when exposed to weathering for a long period of time. If permanently exposed, the lifetime of these materials (often measured by the deterioration of their tensile behaviour) can range from a few months to a few years [10,11,15,16,17,24]. Field weathering tests on an unstabilised PP geotextile revealed that degradation occurs relatively fast, with a reduction in tensile strength close to or greater than 50% after 6 months [10,11]. By month 12, the unstabilised PP geotextile was extremely damaged (reduction in tensile strength of around 94% or more) [10,11]. For stabilised materials, degradation is slower. In a previous work [10], reductions in tensile strength ranging from 39 to 82% were reported for three PP geotextiles with different stabilisation packages exposed to natural weathering for 36 months. In another work [15], a PP geotextile experienced a reduction in tensile strength of 84%, also after 36 months.

In many applications, geotextiles are exposed to UV radiation and other weathering agents for a short period of time, corresponding to the time required for installation activities. Indeed, to perform some functions (e.g., separation, filtration or drainage) the materials must be covered. To prevent premature failure, the uncovered use of these materials is often limited by setting a maximum allowable time (which can be determined from the results of an artificial weathering test, such as EN 12224 [12]). In some cases (e.g., in reinforcement applications), the mechanical properties of geotextiles can be affected by reduction factors to account for degradation. There are some cases, for example in reservoirs, where the materials can be exposed to weathering for long periods of time, sometimes for their entire service life. In this situation, laboratory tests such as EN 12224 [12] do not provide information that allows estimating the lifetime of geotextiles. Laboratory methods to simulate long-term weathering in a short period of time are not available. The existing methods can be extended, and their conditions modified to represent more demanding scenarios, but establishing laboratory–field relationships has proven to be hard to accomplish.

This work studies the weathering resistance of a nonwoven PP geotextile, both under natural and accelerated degradation conditions. The main goals of the work included: (1) determine not only how the tensile properties of the geotextile were affected by natural weathering, but also how other mechanical properties, as well as physical and hydraulic properties, were affected, and (2) compare the degradation suffered by the geotextile outdoors and in the laboratory, looking for relationships between natural and artificial weathering.

## 2. Materials and Methods

The experimental campaign of this work included subjecting a geotextile to weathering under natural degradation conditions. After that, the physical (mass per unit area and thickness), mechanical (tensile, tearing and static puncture behaviour) and hydraulic (water permeability normal to the plane) properties of the geotextile (exposed samples) were evaluated and compared to those obtained before degradation (unexposed sample). Scanning electron microscopy (SEM) was used to analyse the nonwoven structure before and after exposure to natural weathering. Laboratory weathering tests (under accelerated degradation conditions) were also conducted, and the results (only tensile strength) were compared to those found under natural degradation conditions.

### 2.1. Geotextile

A nonwoven geotextile (designated as GT500 in this article) was studied in this work. GT500 was made by needle punching using PP fibres stabilised with two chemical additives: Chimassorb 944 (a hindered amine light stabiliser) and carbon black (a pigment that can also protect polymers from UV attack). Chimassorb 944 and carbon black were present in the PP fibres with the mass percentages of 0.2% and 1.08%, respectively. Accordingly, the fibres had a PP mass percentage of 98.72%. The manufacturer stated that GT500 had a mass per unit area of 500 g·m^−2^. GT500 was anisotropic, with its tensile and tearing properties being direction-dependent (tensile and tearing strength were higher in the machine direction of production than in the cross-machine direction of production).

GT500 was supplied as a roll and the procedures for sampling and preparation of test specimens were conducted in accordance with the guidelines of EN ISO 9862 [26]. Specimens were taken for two purposes: (1) for exposure to weathering (in the field and in the laboratory), and (2) for physical, mechanical and hydraulic characterisation of GT500 before weathering. The specimens exposed to weathering tests were also later characterised by physical, mechanical and hydraulic tests.

### 2.2. Field Weathering Tests

GT500 was exposed to weathering under natural degradation conditions in Portugal. The latitude and longitude coordinates of the exposure site were, respectively, 41°13′ N and 8°39′ W. The altitude was 49 metres. GT500 was installed in an exposure stand facing south with an inclination of 30°. The tests lasted for 24 months, with samples collected for characterisation at 6, 12, 18 and 24 months.

The air temperature, solar radiant energy (between 300 and 3000 nm), precipitation and relative humidity were registered during the outdoor exposure (Table 1). For the different exposure periods, the data shown in Table 1 correspond to average values of air temperature and relative humidity (T_Air_ and RH, respectively) and accumulated values of solar radiant energy (*E*), UV radiant energy (*E*_UV_) and precipitation (P). Based on the intervals given by EN 13362 [27] and Greenwood et al. [3], which are respectively 6–9% and 5–10%, UV radiant energy has been estimated to be 7.5% of solar radiant energy—7.5% corresponds to the average value of both intervals.

Two types of specimens were prepared for the field weathering tests: (1) type I, with a width of 200 mm and a length of 300 mm. The top and bottom 100 mm (in length) were used for gripping to the exposure stand and were protected from weathering. These specimens were intended for subsequent tensile tests; (2) type II, which had a width of 250 mm and a length of 400 mm—exposed length of 300 mm (the top and bottom 5 mm were used for gripping to the exposure stand). At the end of the outdoor exposure, the type II specimens were reduced in size (by cutting) in order to have adequate dimensions for mass per unit area, thickness, tearing, static puncture or water permeability normal to the plane tests. They were also used to collect specimens for SEM analysis.

### 2.3. Laboratory Weathering Tests

GT500 was exposed to artificial weathering in a testing equipment from Q-Panel Lab Products (Westlake, OH, USA)—the QUV Weathering Tester, model QUV/spray. This apparatus allows exposing materials to weathering cycles composed of three steps: exposure to UV radiation, simulating the effect of sunlight (UV step), water spray and condensation (these last two simulating the effect of rain and moisture). The UV and condensation steps are conducted at elevated temperatures in order to accelerate the degradation process and, thereby, allow results to be obtained in a relatively short period of time.

The laboratory weathering tests involved the exposure of GT500 to UV radiation, water spray and condensation. UV radiation was provided by UVA-340 lamps. The water used in the spray step was treated microbiologically, purified by reverse osmosis and, finally, deionised in ionic exchange columns. Water was sprayed at room temperature with a flow of 5 L·min^−1^. The condensation step used water from the public supply network, which upon heating produced water vapour that condensed on the surface of the exposed specimens. Both the water spray and condensation steps were carried out in the dark, i.e., the UVA-340 lamps were turned off.

Two different tests were performed using GT500: (1) tests following, as closely as possible, the method described in EN 12224 [12], and (2) adapted tests. The main characteristics of the weathering cycles used in those tests can be seen in Table 2.

GT500 was exposed to around 70 weathering cycles, each lasting for 5 h and 10 min, during the 362 h of the EN 12224 [12] test. Each weathering cycle was composed of a UV step (5 h at 50 °C) followed by a water spray step of 10 min. The duration of the water spray step was shorter than that specified in EN 12224 [12], which is 60 min. This change was necessary due to the water purification system, which was not able to produce enough water for continuous spraying for 60 min—good quality water is needed to avoid clogging the sprinklers of the weatherometer. At a flow of 5 L·min^−1^, 60 min of spray would require 300 L of purified water every 5 h (time period corresponding to the UV step interspersed with the water spray step). This modification in the EN 12224 [12] test conditions allowed a reduction in water consumption with a low expected impact on the results since it is well-known that UV radiation is the most harmful weathering agent for plastic materials. The UVA-340 lamps operated with an irradiance of 0.68 W·m^−2^. The total radiant energy during the 362 h of testing was 50 MJ·m^−2^ between 290 and 400 nm, which is the value specified in EN 12224 [12].

Regarding the adapted tests, GT500 was exposed to a weathering cycle for different periods of time, namely 500, 1000, 2000 and 4000 h. As shown in Table 2, the weathering cycle was composed of a UV step (4 h at 60 °C), followed by a water spray step (10 min) and a condensation step (4 h at 45 °C). As in the EN 12224 [12] test, the UVA-340 lamps operated with an irradiance of 0.68 W·m^−2^. The total radiant exposure at 340 nm (*E*_340nm_) and the total UV (290–400 nm) radiant exposure, *E*_UV_, increased with the increase of the test duration (greater number of weathering cycles, N), as can be observed in Table 3.

The specimen holders of the weatherometer had an area to expose specimens 80 mm wide by 200 mm long. This way, the specimens used in the laboratory weathering tests had a width of 50 mm and a length of 400 mm—the top and bottom 100 mm, in length, were not exposed. These dimensions are compatible with tensile tests according to EN 29073-3 [28]—the resistance of GT500 to artificial weathering was only evaluated by monitoring changes in its tensile behaviour.

### 2.4. Evaluation of the Damage Suffered by GT500

Following the weathering tests, the physical, mechanical and hydraulic properties of GT500 were determined. In addition, a microscopic analysis of GT500 was performed. The results obtained for the weathered samples were compared with those found for an unexposed sample (intact) and, based on the changes that occurred in the properties of GT500, the damage caused by weathering was evaluated. The physical, mechanical and hydraulic properties of GT500 were determined following standard methods (Section 2.4.1, Section 2.4.2, Section 2.4.3, Section 2.4.4, Section 2.4.5 and Section 2.4.6). The number of specimens used in each test was defined by the respective test standard. Furthermore, in all characterisation tests, and for each weathering period, fresh specimens were used, i.e., there was no reuse of specimens in the non-destructive tests.

The results (average values of at least 5 or 10 specimens, as will be indicated below) are presented with 95% confidence intervals. In addition, some results are expressed in terms of variation: ∆X, where X generically represents a property. ∆X was determined in accordance with Equation (1):

(1) $$∆X=\frac{X_{\left(\mathrm{Exposed}\right)}-X_{\left(\mathrm{Unexposed}\right)}}{X_{\left(\mathrm{Unexposed}\right)}}\times \;100$$

where X_(Unexposed)_ and X_(Exposed)_ is a property of GT500 obtained, respectively, before and after the weathering tests. Using Equation (1), variations were determined for mass per unit area (∆µ_A_), thickness (∆*t*), tensile strength (∆T), tearing strength (∆F_R_), puncture strength (∆F_P_) and velocity index for a head loss of 50 mm (∆V*_H_*_50_).

#### 2.4.1. Mass per Unit Area Tests

Mass per unit area (µ_A_, in g·m^−2^) was determined by measuring and weighing square specimens (side of ≈ 100 mm) of GT500. The specimens were measured with a calliper and weighed on an AND (Tokyo, Japan) balance (model HF 300G). For each sample, at least ten specimens were tested. The tests followed the guidelines of EN ISO 9864 [29].

#### 2.4.2. Thickness Tests

Thickness tests were conducted according to EN ISO 9863-1 [30]. Thickness (*t*, in mm) was measured as the distance between a reference plate (where the specimen was placed) and the contacting face of a circular presser foot. For each sample, a minimum number of ten specimens was tested (square specimens with a side of 100 mm). The pressure exerted on the specimens was 2 kPa. A Karl Schröder KG (Weinheim, Germany) testing equipment was used in the thickness tests.

#### 2.4.3. Tensile Tests

Two different methods were used in the tensile tests, depending on the origin of the samples. For the samples exposed to natural weathering, tensile tests were carried out following the EN ISO 10319 [31] method. The EN 29073-3 [28] method was used for the samples exposed to artificial weathering. The need for two methods was due to the characteristics of the laboratory weatherometer, whose specimen holders did not allow using specimens with the same dimensions as those used in the field weathering tests.

**Figure 1:**
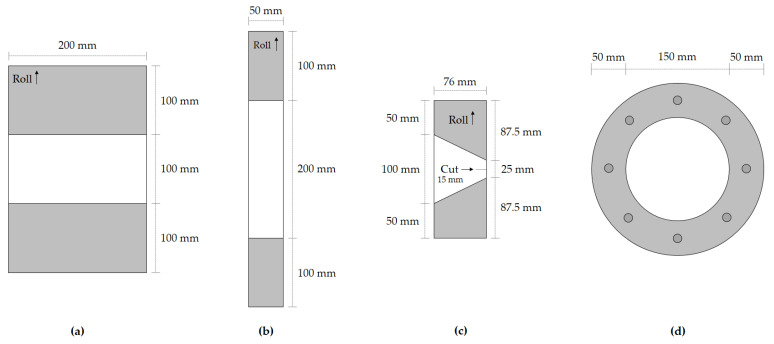
Schematic representation of the specimens used in: (**a**) tensile tests according to EN ISO 10319 [31]; (**b**) tensile tests according to EN 29073-3 [28]; (**c**) tearing tests; (**d**) static puncture tests. The roll arrows indicate the machine direction of production. The gripping area is indicated in grey.

The tensile tests were performed in a Lloyd Instruments (Bognor Regis, UK) testing machine (model LR 50K). These tests were carried out at different displacement rates, depending on the testing method: 20 and 100 mm·min^−1^ for the EN ISO 10319 [31] and EN 29073-3 [28] methods, respectively. In both methods, at least five specimens from each sample were tested (specimens tested in the machine direction of production). The specimens that were subjected to the EN ISO 10319 [31] method had a length of 100 mm (between grips) and a width of 200 mm. In the other method, the specimens were 200 mm long (between grips) and 50 mm wide. Figure 1 schematically represents the specimens used in the tensile tests, as well as those used in the tearing (Section 2.4.4) and static puncture (Section 2.4.5) tests.

Tensile force (F, in N) and elongation were continuously monitored during the tensile tests. Tensile strength (T, in kN·m^−1^), i.e., the maximum tensile force per unit width, was calculated by Equation (2):

(2) $$T=\frac{F_{\max}}{1000}\times \frac{1}{B}$$

where F_max_ corresponds to the maximum tensile force (in N) and B is the width (in m) of the specimen. Elongation at tensile strength (E_T_, in %) was also an output of the tensile tests. Regardless of the tensile test method, elongation was measured based on the relative displacement of the grips, representing the percentage increase in the length of the specimens in relation to their original length (i.e., the initial distance between grips).

#### 2.4.4. Tearing Tests

The tearing tests were carried out according to ASTM D4533 [32] on the same testing machine used for the tensile tests. For each sample, at least ten specimens (in the machine direction of production) were tested. The specimens were rectangular (76 mm wide and 200 mm long) and had an isosceles trapezoid (25 and 100 mm, respectively, at the top and at the base) marked on the centre (area between grips) (Figure 1c). Before the test, a 15 mm cut was made in the middle of the 25 mm side (cut perpendicular to the parallel sides of the trapezoid). Test velocity was 300 mm·min^−1^. Tearing strength (F_R_, in N), i.e., the maximum tearing force, was the property determined in the tearing tests.

#### 2.4.5. Static Puncture Tests

The static puncture tests followed the guidelines of EN ISO 12236 [33] and were conducted on the same testing machine used for the tensile and tearing tests. In these tests, a plunger (stainless steel cylinder with a diameter of 50 mm) was pushed through circular specimens (diameter of 150 mm between grips) at a velocity of 50 mm·min^−1^ (Figure 1d). For each sample, a minimum number of five specimens was tested. The properties determined in the static puncture tests were puncture strength (F_P_, in kN) (maximum puncture force) and push-trough displacement at maximum force (h_P_, in mm).

#### 2.4.6. Water Permeability Normal to the Plane Tests

**Figure 2:**
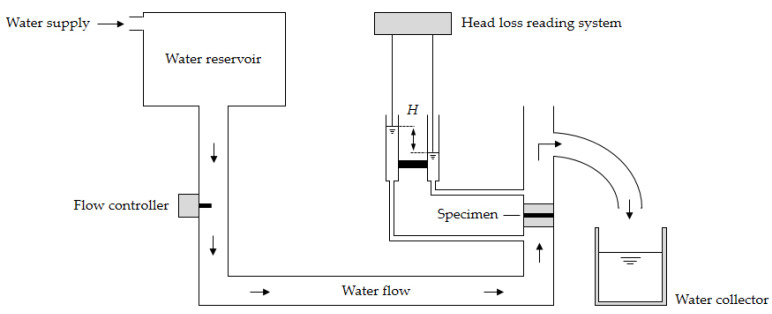
Schematic representation of the equipment used in the water permeability normal to the plane tests (the water course is indicated by arrows).

The water permeability normal to the plane tests were carried out according to the constant head method of EN ISO 11058 [34]. The specimens (five for each sample) were submitted to a unidirectional flow of water under a series of constant head losses, *H*—14, 28, 42, 56 and 70 mm. Circular specimens with a useful diameter (area exposed to the flow of water) of 83.5 mm were used. The tests were conducted on prototype equipment developed at the Faculty of Engineering of the University of Porto, Portugal, in accordance with the instructions of EN ISO 11058 [34] (Figure 2). The equipment had the following main components: a water reservoir, a piping system, a flow controller, a head loss reading system, a specimen mount and a water collector. The internal diameter of the piping system was 83.5 mm.

The hydraulic property obtained in the test was the velocity index for a head loss of 50 mm (V*_H_*_50_, in mm·s^−1^). To calculate V*_H_*_50_, the velocity index at 20 °C (*v*_20_, in mm·s^−1^) was first determined for each of the head losses *H*. The determination of *v*_20_ followed Equation (3), where V is the volume of water (in mm^3^) collected during the time interval t (in s), R_T_ is a correction factor for a water temperature of 20 °C (R_T_ calculated as indicated in EN ISO 11058 [34]) and A is the exposed area (in mm^2^) of the specimens.

(3) $$v_{20}=\frac{V\;R_{T}}{A\;t}$$

The head losses *H* (from 14 to 70 mm) were plotted as a function of the respective *v*_20_ and a quadratic curve that passed through the origin of the graph was fitted to the data. The V*_H_*_50_ was obtained by interpolation in the quadratic regression curve.

#### 2.4.7. Scanning Electron Microscopy

The SEM analyses were conducted on a JEOL (Tokyo, Japan) electronic microscope (model JSM 6310F) equipped with a secondary electron detector. The specimens of GT500 (area of about 1 cm^2^) were metallized with a layer of gold to make them electrically conductive.

## 3. Results and Discussion

### 3.1. Natural Weathering

#### 3.1.1. Physical Properties

The colour of GT500, originally black, did not change significantly over the 24 months of field exposure. However, GT500 became noticeably stiffer, which can be explained by the accumulation of dirt (small particles, e.g., dust, brought by the wind or rain) on its nonwoven structure. The dimensions of the exposed specimens remained practically unchanged. Table 4 summarizes the results obtained for the mass per unit area and thickness of GT500 before and after the field weathering tests.

An analysis of Table 4 shows that the mass per unit area of GT500 had no relevant changes after 6 months (∆_µA_ of +1.4%). However, for longer periods, an increase in mass per unit area was observed (maximum ∆_µA_ of +15.4% after 18 months). This may seem like an odd result, but it can be explained by the dirt that had accumulated on the nonwoven structure of GT500, which is inevitably being taken into account in the mass per unit area test. Therefore, this increase should be seen as a “false increase” as it obviously does not represent a real gain in polymeric mass. It is also relevant to mention that the dirt did not accumulate on top of the specimens but filled the empty spaces that existed between the PP fibres.

Like the mass per unit area, the thickness of GT500 also increased after natural weathering. The increases were relatively small, ranging between 4.3 and 10.1%, with no clear relationship between the magnitude of the increase and the exposure time. The reason for the increase in thickness is the same as previously mentioned for the increase in mass per unit area. The accumulation of dirt on the nonwoven structure has made the material less compressible and, as thickness is determined by applying pressure (in this case, 2 kPa), this resulted in greater thickness.

#### 3.1.2. Mechanical Properties

The exposure to natural weathering also had an impact on the mechanical properties of GT500. However, the changes that occurred varied with the exposure time and did not occur on the same scale for all properties. The tensile, tearing and puncture properties of GT500, before and after the field weathering tests, can be seen in Table 5.

The tensile strength of GT500 (∆T of +1.2%) was practically unchanged after 6 months of natural weathering. The increase in exposure time from 6 to 12 months resulted in a decrease in tensile strength (∆T of −18.6%). Further decreases, namely of 33.3 and 30.2%, were observed after 18 and 24 months, respectively. Comparing these last two periods, the average value of tensile strength was slightly higher after 24 months than after 18 months. However, the dispersion associated with the value obtained for 18 months was relatively high, as shown in Table 5. The elongation at tensile strength of GT500 decreased after all exposure times, with the greatest reduction (from 116.8 to 36.8%) being found after 24 months. As will be seen in Section 3.1.4, SEM analysis will contribute to explaining the reason for the degradation of the tensile behaviour (and also the tearing and puncture behaviour) of GT500.

Like tensile strength, the tearing strength of GT500 also experienced relevant changes during outdoor exposure. However, the variations found in tearing strength had some differences compared to those observed in tensile strength, showing that these properties were affected differently by weathering. Tearing strength experienced a considerable and rapid decrease in the early months of exposure. Indeed, just after 6 months, this property was reduced by almost half (∆F_R_ of −46.7%). Increasing the exposure time to 12 months led to a more marked reduction in tearing strength (∆F_R_ of −61.0%). However, after 12 months, no further meaningful decreases were observed (∆F_R_ of −59.4 and −64.7% after 18 and 24 months, respectively).

The puncture strength of GT500 was also affected by the outdoor exposure, as were the tensile and tearing strengths. This property was almost unchanged after 6 months (∆F_P_ of −3.6%) but changes were noticeable after 12 months (∆F_P_ of −24.3%). The increase in the exposure time tended to result in further, although not very pronounced, reductions in puncture strength (∆F_P_ of −33.1% after 24 months). The push-trough displacement at maximum force of GT500 also decreased after weathering, with the highest reduction (28.3%) found after 24 months.

**Figure 3:**
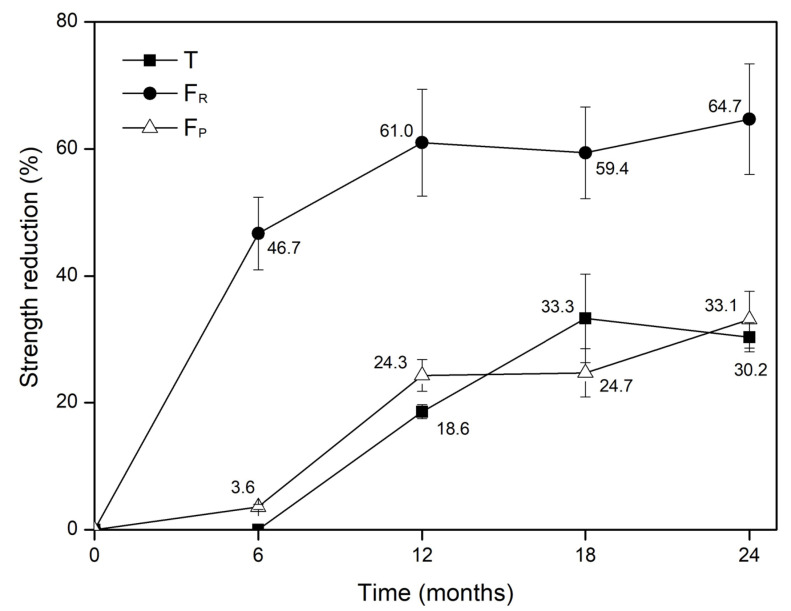
Reduction (in percentage) of mechanical strength over time. (Notation: T—tensile strength; F_R_—tearing strength; F_P_—puncture strength).

Contrary to the physical characterisation tests, the mechanical tests revealed that the outdoor exposure induced some damage to GT500, which resulted in the deterioration of its mechanical behaviour. The changes found in the tensile, tearing and puncture strengths of GT500 were not the same. As illustrated in Figure 3, which compares the variations in the previous properties over time, tearing strength was much more affected than tensile or puncture strengths. Indeed, after 6 months, a 46.7% reduction in tearing strength had already occurred, while the other two properties had no remarkable changes compared to their original values (∆T and ∆F_R_ of, respectively, +1.2 and −3.6%). For longer exposure times, the reductions observed in tearing strength were always higher than those found in the tensile or puncture strengths. Another important conclusion to be drawn from Figure 3 is that, with some relatively small differences, the reductions in tensile strength tended not to be very different from those in puncture strength. Indeed, these properties were more or less identically affected by natural weathering. This result agrees with that observed by Carneiro and Lopes [10]. It also corroborates the existence of a relationship between the tensile strength and puncture strength of nonwoven geotextiles, as reported by Cazzuffi et al. [35]. However, GT500 was anisotropic and the relationship between these two properties did not follow the empiric equation proposed by Cazzuffi et al. [35], which is intended for isotropic nonwoven geotextiles and indicates that tensile strength (in kN·m^−1^) can be estimated by multiplying puncture strength (in N) by 2π. As additional information, it should be mentioned that this equation involves puncture strength values determined according to the method described in EN ISO 12236 [33].

#### 3.1.3. Hydraulic Properties

**Figure 4:**
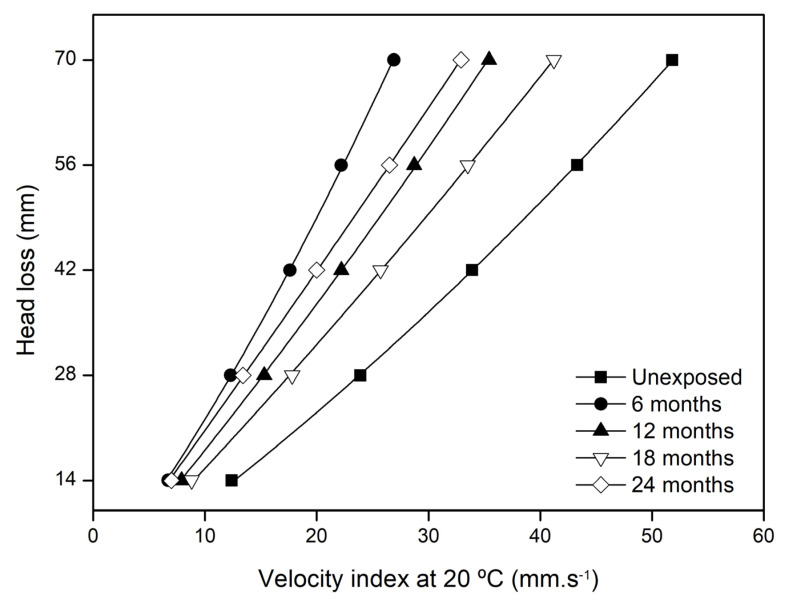
Mean curves head loss vs. *v*_20_ of GT500, before and after the field weathering tests.

As with the previous physical and mechanical properties, the water permeability behaviour of GT500 was also affected by natural weathering. Figure 4 illustrates the mean quadratic curves *H* = f (*v*_20_) obtained for GT500 before and after the field weathering tests. The values found for V*_H_*_50_ are summarised in Table 6.

The exposure to natural weathering had a significant impact on the V*_H_*_50_ of GT500. After 6 months, the value of this property was 20.3 mm·s^−1^, configuring a decrease to almost half (∆V*_H_*_50_ of −48.5%) of its original value. The tests with higher exposure times also induced variations in V*_H_*_50_, but in all cases the values obtained were lower than the original value (∆V*_H_*_50_ between −23.4 and −39.8% for the samples exposed for 12, 18 and 24 months). As can be concluded from the analysis of Table 6, there was no relationship between the increase in exposure time and the change in V*_H_*_50_.

The reduction in the V*_H_*_50_ of GT500 can be explained by the dirt present in its nonwoven structure. Dirt accumulated over time can fill or clog the free spaces between the fibres of nonwoven geotextiles, and hence make it more difficult, or even prevent, the flow of water. Therefore, the accumulated dirt, while not expected to cause chemical or biological damage to the polymeric structure, can significantly affect the water permeability behaviour normal to the plane of nonwoven geotextiles. This way, weathering can influence the ability of these materials to act as filters. In this case, it may be relevant to study the behaviour of nonwoven geotextiles for shorter exposure times (e.g., 1 month), compatible with the expected outdoor exposure in most applications where the materials have a filter function.

#### 3.1.4. SEM Analysis

**Figure 5:**
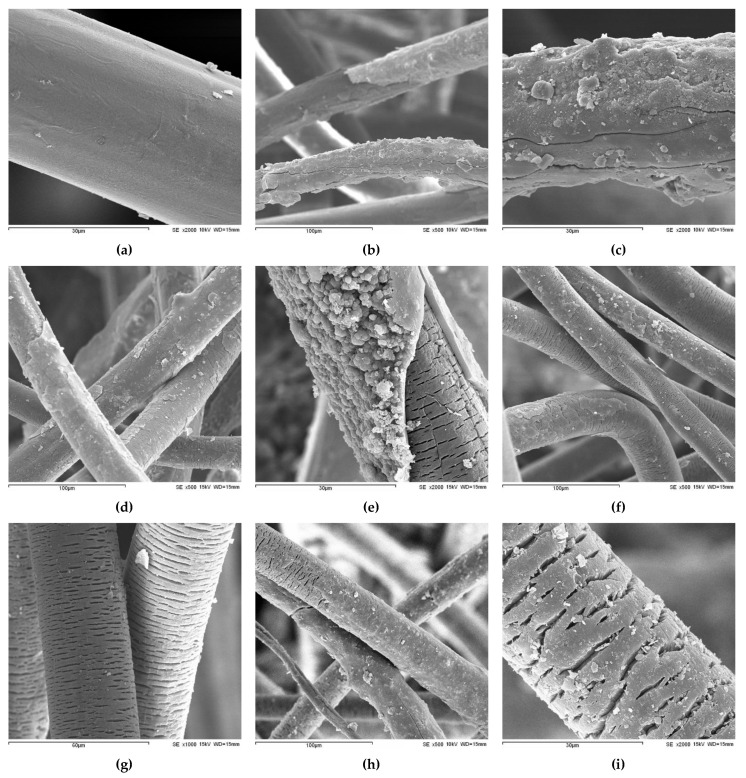
PP fibres of GT500 before and after natural weathering: (**a**) unexposed (2000×); (**b**) 6 months (500×); (**c**) 6 months (2000×); (**d**) 12 months (500×); (**e**) 12 months (2000×); (**f**) 18 months (500×); (**g**) 18 months (1000×); (**h**) 24 months (500×); (**i**) 24 months (2000×). The magnifications correspond to original on-screen values.

In addition to monitoring changes in its physical, mechanical and hydraulic properties, the effect of natural weathering on GT500 was also analysed by SEM. As can be seen in Figure 5b,c, the PP fibres of GT500 were covered with dirt after 6 months, which prevented their observation. For this reason, it was not possible to conclude if they had damage that could explain the decrease found in the tearing strength (∆F_R_ of −46.7%) of GT500. It should be noted that the longitudinal cracks shown in Figure 5b, and in greater detail in Figure 5c, are in the layer of dirt and not in the PP fibres.

The PP fibres were still mostly covered with dirt after 12 months (Figure 5d,e). However, the amount of dirt was low in some areas, making it possible to observe the PP fibres and detect some transverse cracks in these. The cracks were very small, with a maximum width of about 0.6 µm and a length which tended not to exceed 5 µm. The cracking of PP fibres, which is a consequence of photo-oxidation, may explain the decrease found in the mechanical strength of GT500 (Section 3.1.2). The layer of dirt covering a PP fibre can be observed in some detail in Figure 5e. In this case, the layer of dirt had a thickness of about 1 to 2 µm.

Compared to 6 and 12 months, the PP fibres were less dirty after 18 months (Figure 5f,g). This may seem contradictory considering that the maximum mass per unit area value of GT500 was observed precisely at 18 months. However, there is no contradiction, as SEM analysis only shows the top surface of GT500 (the area directly exposed to weathering)—the nonwoven structure had, before weathering, a thickness of 3.68 mm. Despite less dirt on the top surface, there was still a lot of dirt accumulated inside the nonwoven structure of GT500, as indicated by the mass per unit area and thickness values. SEM analysis also showed the existence of many transverse cracks in the PP fibres (Figure 5f,g). These cracks had different lengths, reaching up to about 25 µm, and their maximum width was around 1.5 µm. Compared to 12 months, the cracks were now larger and more abundant.

The SEM images obtained after 24 months of natural weathering (Figure 5h,i) showed that the existing cracks in the PP fibres were not much different from those found after 18 months. Indeed, they had a maximum width of about 1.5–2 µm and a length of up to ≈23 µm. The mechanical results obtained after 18 and 24 months (Section 3.1.2) also tended not to be very different. It is interesting to note that, as observed in previous works [9,15,24] for different geotextiles and exposure conditions, the cracks in the PP fibres were always transverse.

### 3.2. Artificial Weathering

Before starting with the results of artificial weathering, it is relevant to make a short analysis of the tensile results obtained for the unexposed sample. As mentioned in Section 2, different tensile test methods were used to evaluate the tensile behaviour of the samples exposed to natural and artificial weathering. Therefore, it was necessary to test the unexposed sample (which provides the reference values for monitoring degradation) by both methods. As it can be seen in Table 5 and Table 7, the values obtained for the tensile strength of GT500 were identical regardless of the tensile test method: 25.58 and 25.65 kN·m^−1^, respectively, when determined by EN ISO 10319 [31] and EN 29073-3 [28]. By contrast, elongation at tensile strength was significantly lower when GT500 was tested according to EN 29073-3 [28]. This difference can be explained by the particularities of the methods, which, among other things, use specimens with different lengths and widths, as described in Section 2.4.3.

In the laboratory, the weathering resistance of GT500 was initially evaluated following EN 12224 [12] as closely as possible. As shown in Table 7, testing according to this method did not lead to meaningful changes in the tensile properties of GT500. Indeed, a ∆T of +4.2% was observed and the corresponding elongation at tensile strength was also practically unaffected.

UV radiation is considered as one of the main damaging agents for plastic materials exposed outdoors. In order to further investigate the weathering resistance of GT500, additional laboratory tests were carried out with higher exposure times (i.e., higher radiant exposures) than those considered in EN 12224 [12]. In order to accelerate the degradation process (it is well known that the velocity of many chemical reactions increases with increasing temperature), the UV step of these tests was performed at 60 °C instead of the 50 °C used in the EN 12224 [12] method (an increase of 10 °C in temperature often results in the duplication of the reaction rate). In addition, a condensation step was also introduced in the weathering cycle.

GT500 had no visible damage after the different modified weathering tests. However, there were some changes in its tensile behaviour (Table 7). The tests with shorter exposure times did not significantly affect the tensile strength of GT500 (∆T of +1.1 and +7.4% after 500 and 1000 h, respectively). Likewise, they also had no impact on its elongation at tensile strength. The increase in the exposure time, namely to 2000 and 4000 h, resulted in a decrease in tensile strength (∆T of −12.0 and −31.8%, respectively). These reductions were accompanied by decreases in elongation at tensile strength.

### 3.3. Natural Weathering vs. Artificial Weathering

In this section, the degradation of GT500 under natural and artificial weathering conditions is compared. The comparison will be based on the changes undergone by its tensile strength, which was a property measured after both weathering tests.

The method described in EN 12224 [12] intends to differentiate materials with little or no resistance to weathering from those that have this resistance. According to this method, GT500 showed good resistance to weathering, without any relevant changes in its tensile behaviour. With this result, and following the guidelines of ISO/TR 20432 [36], it would be acceptable to expose GT500 outdoors for at least 1 month, without the need to apply any reduction factor to allow for weathering. As shown by the results obtained under natural weathering (Table 5), GT500 maintained its tensile strength for at least 6 months. Indeed, a ∆T of +1.2% was observed after 6 months, with only a reduction in tensile strength found after 12 months (∆T of −18.6%). This shows that, in the case of GT500, the exposure conditions and test results of EN 12224 [12] were able to ensure its correct behaviour, in terms of tensile strength, for 6 months.

**Figure 6:**
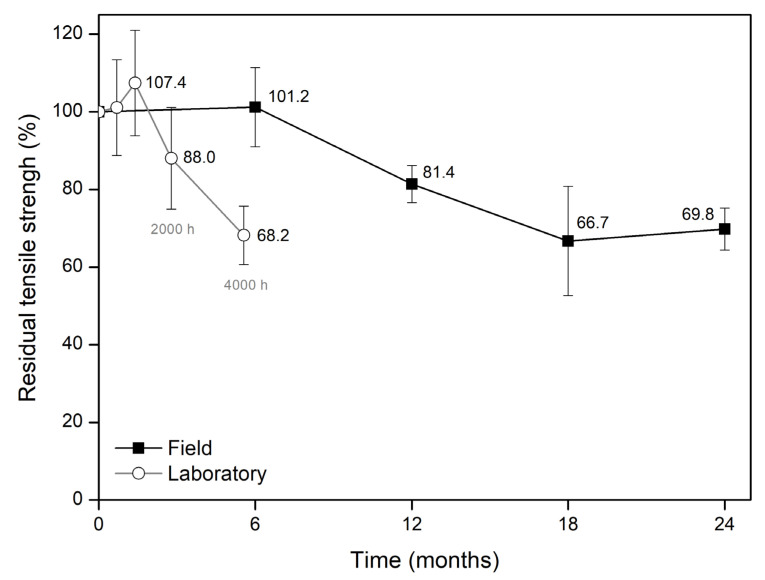
Comparison of the residual tensile strength of GT500 after the field and modified laboratory weathering tests.

Regarding the adapted laboratory weathering tests, the analysis of the residual tensile strengths of GT500 shows that some relationship can be found between natural and artificial weathering (Figure 6). Residual tensile strength (in %) was, regardless of the tensile test method, obtained by dividing the tensile strength of the exposed samples by the tensile strength of the unexposed sample.

The change in tensile strength caused by 12 months outdoors (∆T of −18.6%) was not very different (only slightly more pronounced) than that occurred after 2000 h of exposure in the laboratory weatherometer (∆T of −12.0%). Doubling the exposure time to 24 months and 4000 h, the changes found in tensile strength were very similar (∆T of, respectively, −30.2 and −31.8%). This showed that 4000 h in the laboratory weatherometer (*E*_UV_ of 276 MJ·m^−2^) was practically equivalent to 24 months outdoors (predicted *E*_UV_ of 711 MJ·m^−2^). The reduction in tensile strength observed after 18 months outdoors (∆T of −33.3%) was also similar to that induced by 4000 h in the laboratory weatherometer. However, it is difficult to establish a reliable relationship based on these results due to the high dispersion associated with the tensile strength value obtained after 18 months outdoors.

When comparing the field and laboratory weathering tests, it is possible to conclude that different UV radiant exposures (outdoors and in the laboratory) resulted in identical changes in tensile strength. For example, the reductions of about 30% in tensile strength found after 24 months and 4000 h of natural and artificial weathering, respectively, support this conclusion. The results also allow us to conclude that, despite having higher UV radiant exposures, the field weathering tests did not have a more pronounced impact on tensile strength than the laboratory weathering tests. This can be illustrated, for example, by comparing the results obtained after 12 months outdoors (predicted *E*_UV_ of 421 MJ·m^−2^) and 4000 h in the laboratory weatherometer (*E*_UV_ of 276 MJ·m^−2^). Indeed, ∆T of −18.6 and −31.8%, respectively, were found after these tests. The faster degradation in the laboratory may be explained by the temperature to which GT500 was exposed, which was predictably higher in the accelerated weathering tests (60 °C in the UV step) than in the field weathering tests (temperature not monitored at the surface of GT500, which had a black colour). In addition, it is possible that the dirt accumulated in the nonwoven structure of GT500 during the outdoor exposure had a positive contribution to retarding degradation by preventing sunlight from reaching the PP fibres.

The relationship found in this work between natural and artificial weathering should be considered with care and should not be generalized or extrapolated directly to other materials. Three reasons can be pointed out for this: (1) the results obtained for GT500, which had a particular stabilisation package, may not be valid for other materials with other stabilisation packages; (2) geotextiles made from other polymers may not have the same behaviour when exposed in the laboratory and outdoors; and (3) a different exposure site, with different weather conditions, may not lead to the same results. Even the same exposure site at different time periods may result in different results. Therefore, obtaining a universal relationship between natural and artificial weathering is a difficult, if not impossible, task.

Despite the difficulty in extrapolating the results to other materials, the outcomes of this work may be useful to help foresee the damage that other PP geotextiles (with a stabilisation package identical to that of GT500) may suffer due to weathering. For this, it will be necessary to account for the different climatic conditions (e.g., UV radiant exposure and temperature) to which the materials are expected to be exposed (weather is not reproducible). The relationship found between natural and artificial weathering may be helpful, based on laboratory test results, in providing an indication of the behaviour of a material under natural degradation conditions. However, any prediction must be made carefully, knowing in advance that the result may not be the most accurate.

## 4. Conclusions

This work evaluated the resistance of a PP geotextile (designated as GT500) to weathering, both outdoors (under natural degradation conditions) and in the laboratory (under accelerated degradation conditions). The damage experienced by GT500 in the weathering tests was assessed by monitoring changes in its physical, mechanical and hydraulic properties, and by microscopic analysis. The main results of the work are as follows:

- The mass per unit area and thickness of GT500 increased (less than 10% in most cases) after the field weathering tests. This was due to the presence of dirt on the nonwoven structure and, obviously, not to an increase in polymeric mass.
- The mechanical properties of GT500 degraded during outdoor exposure. The percentage reductions observed in tensile and puncture strength were not very different from each other (around 30% after 24 months) but were less pronounced than those found in tearing strength (around 65% after 24 months).
- The water permeability normal to the plane of GT500 was affected by natural weathering, with the material becoming less permissive—reductions in the velocity index for a head loss of 50 mm ranging from 23.4 to 48.5%. This can be attributed to the dirt existing in the nonwoven structure, which filled the free spaces between the PP fibres and reduced the flow of water.
- SEM analysis showed that outdoor exposure induced transverse cracks in the PP fibres, which may explain the deterioration in the mechanical behaviour of GT500. It also showed the presence of dirt on the nonwoven structure.
- According to the method of EN 12224 [12], GT500 had adequate resistance to artificial weathering, without changes in its tensile behaviour. Compared to outdoor results, the test conditions and results of this method were able to ensure, in terms of tensile strength, the correct behaviour of the material for 6 months.
- The adapted laboratory weathering tests, with exposure conditions more severe than those of the EN 12224 [12] method, caused the deterioration of the tensile behaviour of GT500. The reduction in tensile strength observed after 4000 h in the laboratory weatherometer (*E*_UV_ of 276 MJ·m^−2^) was very close to that found after 24 months outdoors (predicted *E*_UV_ of 711 MJ·m^−2^). This relationship may not be valid for other geotextiles (e.g., made from different polymers or with different stabilisation packages) or other exposure locations.

Overall, GT500 showed some resistance to weathering. For most applications, where the exposure time to UV radiation and other weathering agents is short (less than 1 month, corresponding to the period required for installation), this type of degradation may not be a problem for a properly stabilised PP geotextile. However, for applications where exposure over a long period of time is expected, weathering can significantly affect the behaviour of geotextiles, even if they are stabilised against UV radiation (as GT500 was). In the latter cases, care should be taken when selecting materials to be applied, and it is essential to ensure that their weathering resistance is in compliance with the expected exposure conditions.

## Figures and Tables

**Table 1:** Climate parameters of the exposure site. (Notation: T_Air_—average air temperature; *E*—accumulated solar radiant energy; *E*_UV_—accumulated UV radiant energy; P—accumulated precipitation; RH—average relative humidity).

Period (Months)	T_Air_ (°C)	*E*^1^ (MJ·m^−2^)	*E*_UV_ ^2^ (MJ·m^−2^)	P (mm)	RH (%)
1–6	14.0	2189	164	318	72.3
7–12	21.6	3423	257	256	70.5
13–18	14.5	534	40	547	74.7
19–24	20.3	3329	250	460	74.5

^1^ Measured between 300 and 3000 nm. ^2^ Obtained by estimate—7.5% of the accumulated solar radiant energy.

**Table 2:** Main characteristics of the weathering cycles.

Test	Weathering Cycle	Temperature (°C)	Duration (Hours)
Standard	Step 1. UV	50	5
	Step 2. Water spray ^1^	-	0.167
Adapted	Step 1. UV	60	4
	Step 2. Water spray	-	0.167
	Step 3. Condensation ^1^	45	4

^1^ After this step, the system returns to step 1.

**Table 3:** The number of weathering cycles and total radiant exposure for the different laboratory weathering tests. (Notation: N—number of cycles; *E*_340nm_—total radiant exposure at 340 nm; *E*_UV_—total UV radiant exposure).

Test	Time (Hours)	N	*E*_340nm_ (MJ·m^−2^)	*E*_UV_ (MJ·m^−2^)
Standard	362	70.1	0.86	50
Adapted	500	61.2	0.60	35
	1000	122.4	1.20	69
	2000	244.9	2.40	138
	4000	489.8	4.80	276

**Table 4:** Physical properties of GT500 before and after the field weathering tests. (Notation: µ_A_ – mass per unit area; ∆µ_A_ – variation in mass per unit area; *t* – thickness; ∆*t* – variation in thickness).

Time (Months)	µ_A_ (g·m^−2^)	∆µ_A_ (%)	*t* (mm)	∆*t* (%)
0	499 ± 27	-	3.68 ± 0.06	-
6	506 ± 14	+1.4	3.84 ± 0.06	+4.3
12	545 ± 25	+9.2	4.02 ± 0.06	+9.2
18	576 ± 31	+15.4	3.92 ± 0.13	+6.5
24	542 ± 23	+8.6	4.05 ± 0.06	+10.1

**Table 5:** Mechanical properties of GT500 before and after the field weathering tests. (Notation: T—tensile strength; E_T_—elongation at tensile strength; F_R_—tearing strength; F_P_—puncture strength; h_P_—push-trough displacement at maximum force).

Time (Months)	T (kN·m^−1^)	E_T_ (%)	F_R_ (N)	F_P_ (kN)	h_P_ (mm)
0	25.58 ± 0.85	116.8 ± 3.3	572 ± 41	2.51 ± 0.19	63.3 ± 0.7
6	25.89 ± 2.47	93.4 ± 6.5	305 ± 30	2.42 ± 0.19	52.7 ± 1.2
12	20.81 ± 1.02	50.6 ± 3.9	223 ± 26	1.90 ± 0.14	47.5 ± 0.4
18	17.07 ± 3.57	50.5 ± 5.1	232 ± 23	1.89 ± 0.26	45.8 ± 2.9
24	17.86 ± 1.25	36.8 ± 1.4	202 ± 23	1.68 ± 0.19	45.4 ± 2.4

**Table 6:** V*_H_*_50_ of GT500 before and after the field weathering tests. (Notation: V*_H_*_50_—velocity index for a head loss of 50 mm; ∆V*_H_*_50_—variation in V*_H_*_50_).

Time (Months)	V*_H_*_50_ (mm·s^−1^)	∆V*_H_*_50_ (%)
0	39.4 ± 5.0	-
6	20.3 ± 6.0	−48.5
12	26.1 ± 7.4	−33.8
18	30.2 ± 9.7	−23.4
24	23.7 ± 8.1	−39.8

**Table 7:** Tensile properties of GT500 before and after the laboratory weathering tests. (Notation: *E*_UV_—total UV radiant exposure; T—tensile strength; E_T_—elongation at tensile strength; ∆T—variation in tensile strength).

Time (Hours)	*E*_UV_ (MJ·m^−2^)	T (kN·m^−1^)	E_T_ (%)	∆T (%)
0	0	25.65 ± 1.46	63.0 ± 2.7	-
362 *	50	26.72 ± 3.14	61.2 ± 5.0	+4.2
500	35	25.93 ± 2.78	59.8 ± 2.7	+1.1
1000	69	27.56 ± 3.13	59.4 ± 6.7	+7.4
2000	138	22.58 ± 3.09	41.9 ± 2.7	−12.0
4000	276	17.50 ± 1.64	38.8 ± 1.5	−31.8

* Test according to EN 12224 [12].

## Data Availability

The data presented in this study are available on request from the corresponding author.

## Notation

A—area; B—width; *E*—solar radiant energy; *E*_340nm_—radiant exposure at 340 nm; E_T_—elongation at tensile strength; *E*_UV_—UV radiant energy; F—tensile force; F_max_—maximum tensile force; F_P_—puncture strength; F_R_—tearing strength; *H*—head loss; h_P_—push-trough displacement at maximum force; N—number of weathering cycles; P—precipitation; RH—relative humidity; R_T_—correction factor for a water temperature of 20 °C; t—time interval; *t*—thickness; T—tensile strength; T_Air_—air temperature; V—volume; *v*_20_—velocity index at 20 °C; V*_H_*_50_—velocity index for a head loss of 50 mm; X—property X (generic); X_(Exposed)_—property X after the weathering tests; X_(Unexposed)_—property X before the weathering tests; ∆F_P_—variation in puncture strength; ∆F_R_—variation in tearing strength; ∆*t*—variation in thickness; ∆T—variation in tensile strength; ∆V*_H_*_50_—variation in the velocity index for a head loss of 50 mm; ∆X—variation in property X; ∆_µA_—variation in mass per unit area; µ_A_—mass per unit area.

## References

[1] Shukla S.K., Shukla S.K. (2002). Fundamentals of geosynthetics. Geosynthetics and Their Applications.

[2] Suits L.D., Hsuan Y.G. (2003). Assessing the photo-degradation of geosynthetics by outdoor exposure and laboratory weatherometer. Geotext. Geomembr..

[3] Greenwood J.H., Schroeder H.F., Voskamp V. (2016). Durability of Geosynthetics.

[4] Allen S.R., Koerner R.M. (2016). Geotextile durability. Geotextiles: From Design to Applications.

[5] Feldman D. (2002). Polymer weathering. J. Polym. Environ..

[6] Zweifel H., Maier R., Schiller M. (2009). Plastic Additives Handbook.

[7] Maier C., Calafut T. (1998). Polypropylene: The Definitive User’s Guide and Databook.

[8] Wright D.C. (2001). Failure of Polymer Products Due to Photo-Oxidation.

[9] Carneiro J.R., Almeida P.J., Lopes M.L. (2011). Accelerated weathering of polypropylene geotextiles. Sci. Eng. Compos. Mater..

[10] Carneiro J.R., Lopes M.L. (2017). Natural weathering of polypropylene geotextiles treated with different chemical stabilisers. Geosynth. Int..

[11] Carneiro J.R., Morais M., Lopes M.L. (2018). Degradation of polypropylene geotextiles with different chemical stabilisations in marine environments. Constr. Build. Mater..

[12] EN 12224 (2000). Geotextiles and Geotextile-Related Products—Determination of the Resistance to Weathering.

[13] ASTM D4355 (2021). Standard Test Method for Deterioration of Geotextiles by Exposure to Light, Moisture, and Heat in a Xenon Arc-Type Apparatus.

[14] ASTM D5970 (2002). Standard Practice for Deterioration of Geotextiles from Outdoor Exposure.

[15] Aparicio-Ardila M.A., Pedroso G.O.M., Kobelnik M., Valentin C.A., Luz M.P., Silva J.F. (2021). Evaluating the degradation of a nonwoven polypropylene geotextile exposed to natural weathering for 3 years. Int. J. of Geosynth. Ground Eng..

[16] Grubb D.G., Diesing W.E., Cheng S.C.J., Sabanas R.M. (2000). Comparison of geotextile durability to outdoor exposure conditions in the Peruvian Andes and the southeastern USA. Geosynth. Int..

[17] Hsieh C., Wang J.-B., Chiu Y.F. (2006). Weathering properties of geotextiles in ocean environments. Geosynth. Int..

[18] Guimarães M.G.A., de Mattos Vidal D., de Carvalho Urashima D., Castro C.A.C. (2017). Degradation of polypropylene woven geotextile: Tensile creep and weathering. Geosynth. Int..

[19] Koerner R.M., Hsuan Y.G., Koerner G.R. (2017). Lifetime prediction of exposed geotextiles and geomembranes. Geosynth. Int..

[20] Valentin C.A., Kobelnik M., Franco Y.B., Lavoie F.L., da Silva J.L., da Luz M.P. (2021). Study of the ultraviolet effect and thermal analysis on polypropylene nonwoven geotextile. Materials.

[21] Franco Y.B., Valentin C.A., Kobelnik M., da Silva J.L., Ribeiro C.A., da Luz M.P. (2022). Accelerated aging ultraviolet of a PET nonwoven geotextile and thermoanalytical evaluation. Materials.

[22] Valente I.M., Carneiro J.R., Almeida P.J., Lopes M.L. Chemical studies about the durability of polypropylene geotextiles. Proceedings of the 9th International Conference on Geosynthetics.

[23] Valente I.M., Carneiro J.R., Almeida P.J., Lopes M.L. (2011). Determination of Chimassorb 944 in polypropylene geotextiles by HPLC-UV. Anal. Lett..

[24] Carneiro J.R., Almeida P.J., Lopes M.L. (2019). Evaluation of the resistance of a polypropylene geotextile against ultraviolet radiation. Microsc. Microanal..

[25] Filho J.L.E.D., de Almeida Maia P.C., de Castro Xavier G. (2019). Spectrophotometry as a tool for characterizing durability of woven geotextiles. Geotext. Geomembr..

[26] EN ISO 9862 (2005). Geosynthetics—Sampling and Preparation of Test-Specimens.

[27] EN 13362 (2013). Geosynthetic Barriers—Characteristics Required for Use in the Construction of Canals.

[28] EN 29073-3 (1992). Textiles—Test Methods for Nonwovens—Part 3: Determination of Tensile Strength and Elongation.

[29] EN ISO 9864 (2005). Geosynthetics—Test Method for the Determination of Mass per Unit Area of Geotextiles and Geotextile-Related Products.

[30] EN ISO 9863-1 (2016). Geosynthetics—Determination of Thickness at Specified Pressures—Part 1: Single Layers.

[31] EN ISO 10319 (2015). Geosynthetics—Wide-Width Tensile Test.

[32] ASTM D4533 (2002). Standard Test Method for Trapezoid Tearing Strength of Geotextiles.

[33] EN ISO 12236 (2006). Geosynthetics—Static Puncture Test (CBR Test).

[34] EN ISO 11058 (2019). Geotextiles and Geotextile-Related Products—Determination of Water Permeability Characteristics Normal to the Plane.

[35] Cazzuffi D., Venesia S., Rinaldi M., Zocca A. The mechanical properties of geotextiles: Italian standards and interlaboratory test comparison. Proceedings of the 3rd International Conference on Geotextiles.

[36] ISO/TR 20432 (2007). Guidelines for the Determination of the Long-Term Strength of Geosynthetics for Soil Reinforcement.

